# Perceptual learning to discriminate the intensity and spatial location of nociceptive stimuli

**DOI:** 10.1038/srep39104

**Published:** 2016-12-20

**Authors:** Flavia Mancini, Karina Dolgevica, James Steckelmacher, Patrick Haggard, Karl Friston, Giandomenico D. Iannetti

**Affiliations:** 1 Department of Neuroscience, Physiology and Pharmacology, University College London, London, UK; 2 Institute of Cognitive Neuroscience, University College London, London, UK; 3 Wellcome Trust Centre of Neuroimaging, University College London, London, UK

## Abstract

Accurate discrimination of the intensity and spatial location of nociceptive stimuli is essential to guide appropriate behaviour. The ability to discriminate the attributes of sensory stimuli is continuously refined by practice, even throughout adulthood - a phenomenon called perceptual learning. In the visual domain, perceptual learning to discriminate one of the features that define a visual stimulus (e.g., its orientation) can transfer to a different feature of the same stimulus (e.g., its contrast). Here, we performed two experiments on 48 volunteers to characterize perceptual learning in nociception, which has been rarely studied. We investigated whether learning to discriminate either the intensity or the location of nociceptive stimuli (1) occurs during practice and is subsequently maintained, (2) requires feedback on performance, and (3) transfers to the other, unpractised stimulus feature. First, we found clear evidence that perceptual learning in discriminating both the intensity and the location of nociceptive stimuli occurs, and is maintained for at least 3 hours after practice. Second, learning occurs only when feedback is provided during practice. Finally, learning is largely confined to the feature for which feedback was provided. We discuss these effects in a predictive coding framework, and consider implications for future studies.

Skin nociceptors are activated by intense mechanical, thermal and chemical stimuli, which signal potentially dangerous objects. Accurate discrimination of both the intensity and the spatial location of these stimuli is essential to determine appropriate motor behavior in everyday life.

The ability to discriminate the attributes of sensory stimuli is continuously refined through practice, even throughout adulthood. This phenomenon, often referred to as perceptual learning^1^, is important to adapt behaviour to the environment. Perceptual learning is typically obtained in an experimental context by providing feedback on discrimination performance, on a trial-by-trial basis^1^. Several mechanisms underlying perceptual learning have been proposed, from refined encoding of sensory information to improved decision making^2^,^3^,^4^,^5^. Interestingly, learning is not necessarily confined to a specific stimulus feature: learning to discriminate one of the features that defines a sensory stimulus (e.g., the orientation of a visual stimulus) sometimes transfers, or generalizes, to a different feature of the same stimulus (e.g., the contrast of the same visual stimulus)^6^,^7^.

Despite a rich tradition of work in the visual, auditory and tactile domains, perceptual learning in the nociceptive system has been rarely studied. It is therefore unknown whether and how learning refines our ability to discriminate the intensity and location of nociceptive input. Given the behavioural importance of appropriate discrimination of nociceptive stimuli, learning should also occur within the nociceptive system.

In this study, we performed two experiments on 48 healthy volunteers, to characterize perceptual learning in nociception. First, we tested whether learning to discriminate either the intensity or the location of nociceptive stimuli occurs following explicit trial-by-trial feedback on performance (supervised practice), and whether it also emerges through passive exposure to the stimulus feature to be discriminated (unsupervised practice). Second, we tested whether learning to discriminate either the intensity or the spatial location of nociceptive stimuli transfers to the other stimulus feature. The feature-specificity of learning is often interpreted as the hallmark that learning modulated early sensory representations that are specific to the practiced feature^3^. In contrast, the generalization of learning across different discrimination tasks is generally interpreted as learning modulated processes that are less or not at all feature-specific^8^.

Both experiments were performed in a blinded fashion: an experimenter unaware of the practice condition assessed discrimination in two sessions: 3 hours before practice (baseline), and 3 hours after practice (+3 h test). Nociceptive stimuli were radiant heat laser pulses (diameter: 6 mm), which selectively activate skin nociceptors^9^, and allow optimal control of both stimulus intensity and spatial location^10^,^11^.

With respect to the occurrence of learning and its dependence on practice effects (i.e. regardless of transfer), in both experiments we compared the effects of verbal feedback versus no feedback on discrimination performance. During supervised practice, participants performed either the intensity discrimination task or the spatial discrimination task while receiving trial-by-trial feedback on performance. Verbal feedback (“correct”, “error”) was only given in the supervised condition, by playing pre-recorded signals. We hypothesized that learning effects would be stronger in the supervised than the unsupervised condition.

With respect to learning transfer, in Experiment 1 we investigated whether learning in the intensity domain transferred to spatial discrimination, and in Experiment 2 we tested whether learning in the spatial domain transferred to intensity discrimination. Given that the transfer of perceptual learning can depend on the perceptual precision of the discrimination task^12^, we designed intensity and spatial discrimination tasks that required comparable levels of precision.

## Results

### Learning during practice

**Figure 1:**
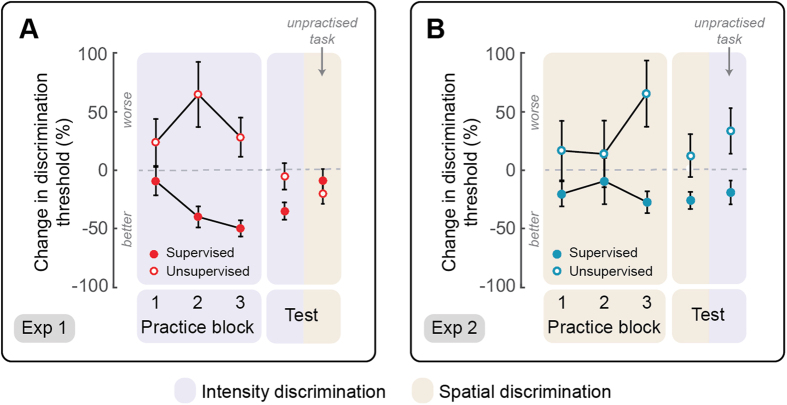
Group results. The plots display the group average (±SE) percentage change of intensity (purple) and spatial (beige) discrimination thresholds during the first three blocks of supervised and unsupervised practice, and at the +3 h test session. Percent changes of discrimination thresholds were calculated relative to baseline (3 h before practice). Panel A shows results from Experiment 1, in which participants practiced intensity discrimination, whereas panel B shows results from Experiment 2, in which participants practised spatial discrimination.

**Figure 2:**
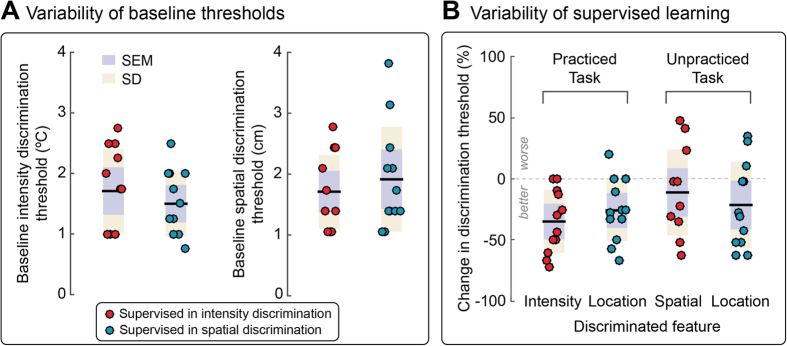
Individual data. (**A**) Intensity and spatial discrimination thresholds estimated at baseline (3 h before practice), in the groups that subsequently received supervised training in intensity and spatial discrimination. (**B**) Percentage change of both intensity and spatial discrimination thresholds, estimated 3 h after supervised practice in intensity and spatial discrimination. Each circle represents an individual participant.

In Experiment 1 (n = 24), one group of participants (n = 12) received feedback while practicing intensity discrimination, whereas a second group (n = 12) did not receive any feedback on performance. In Experiment 2 (n = 24), one group of participants (n = 12) was supervised while practicing spatial discrimination, whereas a second group (n = 12) was unsupervised during practice. We calculated the percentage change of discrimination thresholds relative to baseline, both during practice and 3 hours after practice (Figs 1 and 2).

#### Experiment 1: Effect of intensity discrimination practice

We first tested whether intensity discrimination thresholds improved across the first three blocks of supervised and unsupervised intensity practice. A mixed-effect ANOVA with a within-subject factor ‘practice block’ (3 levels: 1–3) and a between-subject factor ‘practice type’ (2 levels: supervised, unsupervised) provided strong evidence for a difference between practice types (main effect of practice type: F_1,21_ = 12.404, p = 0.002, η^2^_p_ = 0.371), no evidence for a difference across practice blocks (main effect of practice block: F_2,42_ = 1.664, p = 0.202, η^2^_p_ = 0.073), and moderate evidence for a practice block x practice type interaction (F_2,42_ = 5.32, p = 0.009, η^2^_p_ = 0.202). To further explore this interaction we performed three post-hoc tests (Bonferroni-corrected α = 0.017), comparing the change in discrimination performance between the two groups, at each time point. In the first block, there was no evidence for a difference between supervised and unsupervised groups (p = 0.304, d = −0.603). However, in the second and third block, we found strong evidence for better performance in the supervised group relative to the unsupervised group (block 2: p = 0.003, d = −1.523; block 3: p < 0.0001, d = −1.878). To obtain an overall estimate of learning during practice, we averaged discrimination thresholds across all practice blocks and compared them against 0, separately for each practice type, using a one-sample t-test (p-values were estimated using a bootstrap procedure with 1,000 iterations and 95% confidence interval). We found strong evidence of improvement in discrimination performance (i.e., learning) during practice for the group that received feedback (mean ±SE, −29.8% ± 6.89%; p = 0.006, Cohen’s d = −1.207), and moderate evidence of a worsening of discrimination performance in the group that did not receive feedback (+46.04% ± 16.52%; p = 0.026, d = 0.749).

#### Experiment 2: Effect of spatial discrimination practice

As for Experiment 1, we tested whether spatial discrimination thresholds improved across the first three blocks of supervised and unsupervised practice. A mixed ANOVA with a within-subject factor ‘practice block’ (3 levels: 1–3) and a between-subject factor ‘practice type’ (2 levels: supervised, unsupervised) did not show evidence for main effects of practice blocks (F < 1) and practice type (F_1,22_ = 3.344, p = 0.081, η^2^_p_ = 0.132). There was, however, strong evidence for an interaction between practice block and practice type (F_2,44_ = 5.89, p = 0.005, η^2^_p_ = 0.211). To further explore this interaction we performed post-hoc tests (Bonferroni-corrected α = 0.017), comparing the change in discrimination performance between the two groups, at each time point. In the first and second blocks, there was no evidence for a difference between supervised and unsupervised groups (block 1: p = 0.219, d = −0.539; block 2: p = 0.698, d = −0.167). However, in the third block, we found strong evidence for better performance in the supervised group relative to the unsupervised group (p = 0.003, d = −1.441). To obtain an overall estimate of learning during practice, we averaged discrimination thresholds across all practice blocks and compared them against 0, separately for the each practice type, using a one-sample t-test. We found weak evidence for learning in the group that received feedback (−18.41% ± 6.68%; p = 0.038, d = −0.750) and no evidence for learning in the group that did not receive feedback (30% ± 22.6%; p = 0.256, d = 0.366).

Altogether, these results indicate that, during practice, nociceptive learning occurs in both intensity and spatial discrimination. However, it only occurs when the practice is supervised.

### Learning at +3 h test

Approximately three hours after practice, we tested both intensity and spatial discrimination thresholds. At this test stage, no feedback on performance was given to either group. This test session allowed us to assess whether learning to discriminate the practiced stimulus feature was still present 3 hours after practice, and, if so, whether it transferred to the unpractised task.

#### Experiment 1: Effect of intensity discrimination practice

We conducted a mixed-effects analysis of variance with a within-subject factor ‘stimulus feature’ (2 levels: practiced, unpractised) and a between-subject factor ‘practice type’ (2 levels: supervised, unsupervised) on the percentage change in intensity and spatial discrimination thresholds relative to baseline. There was no evidence for main effects of ‘stimulus feature’ (F < 1) or ‘practice type’ (F < 1), and weak evidence for an interaction between ‘stimulus feature’ and ‘practice type’ (F_1,22_ = 4.759, p = 0.040, η^2^_p_ = 0.178). Post-hoc tests provided moderate evidence for a difference in discriminating the practiced and unpractised stimulus feature in the group that was supervised during practice (p = 0.021, d = −0.867), and, as expected, no evidence for a difference in discriminating the practiced and unpractised stimulus feature in the unsupervised group (p = 0.38, d = 0.254).

To check whether learning was still present three hours after supervised practice, we compared the improvement in discrimination performance against 0, using one-sample t-tests (i.e., no change relative to baseline). In the group that was supervised during practice, we found strong evidence of learning in the practiced intensity discrimination task (p = 0.002, d = −1.358), but not in the unpractised spatial discrimination task (p = 0.387, d = −0.255). In contrast, in the group that was not supervised during practice, we found no evidence of learning in intensity discrimination (p = 0.653, d = −0.133), and weak evidence of learning in spatial discrimination (p = 0.049, d = −0.635).

These findings show that supervised learning to discriminate nociceptive intensity is maintained for at least 3 hours, and does not transfer to the spatial discrimination of nociceptive stimuli.

#### Experiment 2: Effect of spatial discrimination practice

Similarly to what performed in Experiment 1, we conducted a mixed-effects analysis of variance with a within-subject factor ‘stimulus feature’ and a between-subject factor ‘practice type’ on the percentage change in intensity and spatial discrimination thresholds estimated 3 hours after spatial practice. There was strong evidence for a main effect of practice type (F_1,22_ = 8.717, p = 0.007, η^2^_p_ = 0.284), and no evidence for a main effect of stimulus feature (F < 1). In contrast to what observed in Experiment 1, there was no interaction between practice type and stimulus feature (F < 1), possibly due to the higher inter-individual variability of discrimination performance (see Fig. 2B). The overall change in discrimination thresholds (i.e. in both spatial and intensity discrimination) was greater in the group that was supervised during practice, than in the group that was not supervised (difference: −45.31% ± 15.35%).

To test whether learning was still present three hours after practice, we compared the mean percentage change in discrimination thresholds against 0 (no change relative to baseline). In the group that was supervised during practice, we found strong evidence of learning in the practiced spatial discrimination task (p = 0.003, d = −1.02), but inconsistent evidence of an improvement in the unpractised intensity discrimination task (p = 0.084, bootstrap n/a; d = −0.548). In contrast, in the group that was unsupervised during practice, we found no evidence of learning in either intensity or spatial discrimination tasks (p > 0.12, d < 0.05).

These findings indicate that supervised learning to discriminate the spatial location of nociceptive stimuli through supervised feedback is maintained for at least 3 hours, and does not appear to transfer to the unpractised feature at group-level.

### Comparison between supervised learning in intensity and spatial discrimination

We also tested whether supervised practice in spatial discrimination led to the same degree of learning as supervised practice in intensity discrimination (Fig. 1). We conducted an independent sample t-test to compare the average percentage change in discrimination thresholds estimated both during and after supervised practice in intensity discrimination (Experiment 1) vs. spatial discrimination (Experiment 2). We found no evidence for a difference in the learning achieved by the groups supervised in spatial discrimination and intensity discrimination (learning during practice: p = 0.289, d = 0.483; learning at + 3 h: p = 0.399, d = 0.372). Thus, learning achieved through supervised practice was comparable in the intensity and spatial domains.

### Control 1: effect of exposure to the stimuli

To test whether the observed differences in learning between supervised and unsupervised practice conditions were due to different exposure to the stimuli^1^, we compared the number of trials that were presented, *during* practice, across the four practice conditions of Experiments 1 and 2 (Fig. 2B). We conducted a one-way ANOVA on the total number of trials used to estimate discrimination thresholds during practice, with a between-subject factor practice type (4 levels: supervised intensity, unsupervised intensity, supervised spatial, unsupervised spatial). This analysis did not reveal any effect of practice type (F < 1), thus ruling out that the observed differences in learning were due to different exposure to the stimuli.

### Control 2: effect of baseline discrimination performance

To assess whether the differences in learning to discriminate the intensity and spatial location of the stimulus through supervised versus unsupervised practice were due to a difference in baseline discrimination performance, we pooled baseline discrimination thresholds from Experiments 1 and 2, separately for the supervised and unsupervised features, and performed independent sample t-tests to compare these baseline thresholds across the group supervised in intensity discrimination, and across the group supervised in spatial discrimination, separately for each task. We did not find evidence for a difference of baseline discrimination thresholds across supervised groups, in either the intensity discrimination task (p = 0.42, bootstrap n/a; d = 0.351) or the spatial discrimination task (p = 0.51, bootstrap n/a; d = 0.286; Fig. 2A). Similarly, we found no evidence for a difference of baseline discrimination thresholds across unsupervised groups, in either task (intensity: p = 0.116, bootstrap n/a; d = 0.697; space: p = 0.853, bootstrap n/a; d = 0.08).

### Control 3: effect of task precision

**Figure 3:**
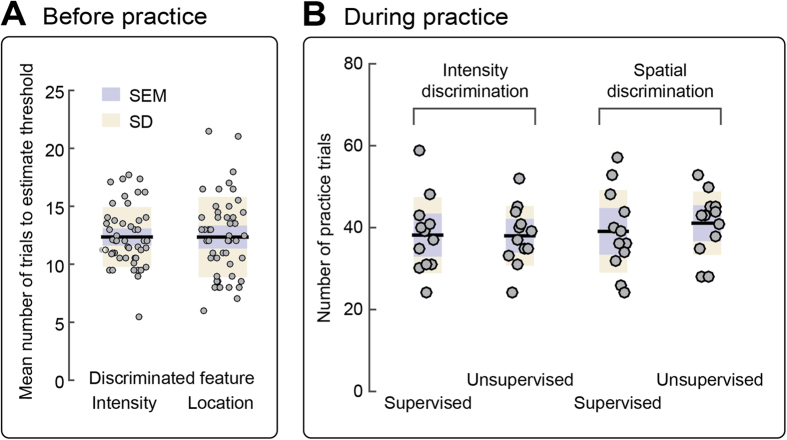
Number of trials necessary to estimate thresholds. (**A**) Mean number of trials used to estimate intensity and spatial discrimination thresholds, at baseline, pooled across practice types. The average number of trials used to estimate intensity and spatial discrimination thresholds were comparable (p = 0.987), suggesting that the precision of intensity and spatial discrimination tasks was balanced. (**B**) The total number of trials presented during supervised and unsupervised practice in intensity and spatial discrimination was comparable across practice types. Each circle represents an individual participant.

Given that the transfer of perceptual learning can depend on the precision of the task^12^, we designed intensity and spatial discrimination tasks that required comparable levels of precision. We quantified the precision of the task as the number of trials required to estimate the discrimination threshold with the method of limits, at baseline. We therefore pooled the average number of these trials across practice types, for each participant. Similarly, we pooled the average number of trials used to estimate spatial discrimination thresholds, at baseline, across practice types. The resulting values are shown in Fig. 3A. The average number of trials used to estimate intensity and spatial discrimination thresholds were comparable (p = 0.987, d = 0.004), suggesting that the precision of intensity and spatial discrimination tasks was balanced.

### Control 4: effect of learning on detection thresholds

Finally, we checked whether detection thresholds of pinprick pain were different at the +3 h testing session, with respect to baseline. We compared the percentage change of pinprick detection threshold at +3 h against 0 (i.e., against no change between baseline and +3 h), separately for each practice type and experiment. There was no evidence for changes in detection thresholds at +3 h in both practice types (Experiment 1, supervised intensity: p = 0.955, d = −0.017; Experiment 1, unsupervised intensity: p = 0.193, d = 0.416; Experiment 2, supervised spatial location: p = 0.932, d = 0.025; Experiment 2, unsupervised spatial location: p = 0.465, d = −0.229).

## Discussion

This study provides the first comprehensive investigation into nociceptive perceptual learning in humans. We obtained three main results. First, the discrimination of the intensity and spatial location of a nociceptive stimulus is improved through perceptual learning. This improvement is maintained for at least 3 h after practice. Second, learning occurs only when feedback is provided during practice. Finally, nociceptive learning in the intensity and spatial domains is largely limited to the practiced feature.

### Learning to discriminate nociceptive stimuli depends on feedback during practice

Although participants were exposed to a comparable number of trials in supervised and unsupervised practice conditions (Fig. 3), nociceptive learning occurred only through supervised practice, both in the intensity and spatial domains (Fig. 1). These results highlight the importance of feedback in learning to discriminate features of sensory stimuli^13^. A few studies have reported that visual learning in a spatial discrimination task can occur in absence of feedback, but of a much smaller degree than in presence of feedback^14^. Importantly, several other studies did not demonstrate consistent visual learning without feedback, even when using the same paradigm^8^,^15^. We cannot exclude that learning could emerge from prolonged unsupervised practice over multiple testing sessions, but we note that we did not observe any evidence of learning in a single unsupervised practice session. Instead of learning, we found moderate evidence of a worsening of discrimination thresholds during unsupervised practice in intensity discrimination, perhaps due to reduced attention in absence of feedback or due to the unpleasant nature of the stimulation.

### Nociceptive learning is largely confined to the practiced stimulus feature

Perceptual learning optimizes processes that are exploited by the practiced discrimination task^3^,^8^,^16^,^17^,^18^. Studies on perceptual learning have shown that transfer of learning is possible, but it is more the exception rather than the rule^3^: the transfer of learning depends on many factors, such as the precision of the tasks^12^, the amount of practice^19^, and the architecture of the neural systems activated by the tasks^2^,^8^. In our experiments, we balanced task precision and amount of practice, and observed that learning to discriminate the intensity and spatial location of nociceptive input is largely confined to the trained feature: it follows that learning is likely to have modulated processes specific to the practiced discrimination tasks.

The information carried by the nociceptive afferent volley is encoded and processed at multiple levels, which include the dorsal horn, the thalamus, the primary and secondary somatosensory cortices (SI and SII) and the dorso-posterior insula^20^,^21^,^22^. All these regions contain somatotopic representations of nociceptive input to the skin^23^,^24^,^25^,^26^,^27^,^28^. However, SI is the only known region containing a fine-grained spatial representation of nociceptive input^23^, which could support the ability to discriminate small changes in the spatial location of nociceptive stimuli^29^. The sensitivity of SI neurons (wide dynamic range neurons) also improves to a point that allows discriminating temperature differences as small as 0.2 °C, in awake monkeys trained to discriminate the intensity of nociceptive stimuli^30^. Thus, it is reasonable to suggest that nociceptive coding in SI is refined through supervised practice in intensity and spatial discrimination. However, it is also possible that representations of nociceptive input in other areas, such as the insula and secondary somatosensory cortices, are also sharpened by supervised discrimination practice.

Functional neuroimaging studies in humans have shown that other higher-order regions, such as the prefrontal, cingulate and parietal areas, are also active during the discrimination of the intensity and spatial location of nociceptive input^31^,^32^,^33^,^34^. Given that learning can occur at multiple processing levels and modulate the inter-regional connectivity, all these areas are potential candidates for experience-dependent plasticity that mediates improvements in perceptual discrimination. We hope that future studies will shed light on the neural correlates of nociceptive learning, which remain largely unknown.

### Effect of exposure to untrained features on learning transfer

Several studies of perceptual learning in the visual domain have reported that exposure to a task-irrelevant sensory feature, during supervised practice on a task-relevant feature, can cause learning to transfer to the task-irrelevant feature. However, learning rarely occurs during unsupervised practice^13^,^35^.

We aimed to minimise the possibility of transfer due to changes in the task-irrelevant feature by selectively manipulating only one sensory feature during the practiced blocks. Indeed, in the intensity discrimination task there was no change in stimulus location: the two successive stimuli in each trial were delivered to the same skin location. Similarly, in the spatial discrimination task, the two stimuli had different locations, but the same intensity. Although we did not observe any consistent transfer of supervised learning at the group level, the inter-individual variability of performance seemed higher in the unpractised (transfer) tasks than in the practiced tasks (Fig. 2B). Our design and sample size do not allow us quantitatively testing inter-individual differences. However, in the interest of future studies, we note that some participants improved in the unpractised task after supervised practice in intensity and spatial discrimination (Fig. 2B).

It is possible that the variability of performance in the unpractised task is due to exposure to small unavoidable changes in the untrained stimulus feature. Indeed, task-irrelevant visual learning occurs only when the discriminability of the task-irrelevant feature is just below discrimination threshold, and only during supervised practice^13^,^35^. In the spatial discrimination task, testing two different skin locations inevitably entailed stimulating two different sets of nociceptive afferents, thus introducing an unavoidable variation in the magnitude of the afferent input. Such unintended exposure to changes in task-irrelevant features might account for the inter-individual variability of learning transfer, particularly from spatial discrimination to intensity discrimination (Fig. 2B).

### Future directions: Interpreting learning in a predictive coding framework

Learning has been interpreted in different computational frameworks, with different emphases on early versus late loci of learning. The predictive coding framework may account for learning at any processing stage, as well as transfer learning. This framework postulates that the brain actively constructs perceptual experiences based on the comparison between predictions of sensory input and the actual sensory input. The idea that the brain tries to infer the causes of its sensations through generative models dates back to the Empiricist tradition, and was first articulated formally by Helmholtz^36^. Hierarchical representations of the hidden or latent causes of sensory input provide descending predictions of the incoming afferent information. These comparisons result in mismatch (or prediction error) signals, which are used to update the representations generating predictions^37^,^38^,^39^,^40^,^41^.

In the predictive coding framework, learning is equivalent to Hebbian plasticity, which minimises prediction error. This applies to perceptual inference or discrimination. Large prediction errors result in greater adjustments to the parameters of models generating sensory predictions. This enables subsequent predictions to minimize prediction errors more efficiently^37^,^42^. When prediction errors are minimal, learning is achieved. Perceptual learning is likely to involve fine-grained representations of the intensity and spatial location of nociceptive stimuli. In this framework, learning can be considered an adjustment of between-areas connectivity and lateral interactions to ensure a more precise representation of sensory input (c.f., representational sharpening) and thereby of the generated sensory impressions. It is likely that the feedback provided during training enhances the effects of prediction errors pertaining to that feature through a mechanism of attentional gain^43^. This hypothesis could be tested by coupling the paradigm described in this study with dynamic causal modelling^44^ of the sources of the electromagnetic responses elicited by nociceptive stimuli during learning.

## Methods

### Participants

Forty-eight healthy volunteers participated in the study (Experiment 1: n = 24, mean age ±SD: 23.08 ± 3.41 years old, 6 males; Experiment 2: n = 24, mean age ±SD: 22.71 ± 3.32 years old, 8 males). All participants gave written informed consent and received payment for their participation. The study was approved by the UCL Research Ethics Committee and was conducted in accordance with the principles of the Declaration of Helsinki.

### Stimuli

Nociceptive stimuli were radiant heat pulses that selectively activate Aδ and C skin nociceptors, without co-activating Aβ mechanoreceptors^45^,^46^. Radiant heat stimuli were generated using a CO_2_ laser (10.6 nm, beam diameter 6 mm), whose power is continuously regulated using a feedback based on an online measurement of skin temperature at the site of stimulation (LSD, SIFEC, Belgium)^47^. This device allows delivering two consecutive stimuli to the same skin location. This characteristic makes it optimal for testing the intensity discrimination: delivering two consecutive stimuli at the same location is important to avoid unwanted exposure to changes in stimulus location during practice in intensity discrimination. Indeed, exposure to changes in a feature other than the feature for which feedback is provided can result in implicit learning^13^. The laser spot size was 6 mm in all tasks, and the stimuli were delivered to a defined 8 × 4 cm skin region in the low back. The decision to stimulate the low back was driven by the observations from a series of pilot experiments, aimed to find a body region where both intensity and spatial discrimination were similarly poor, thus allowing to observe an improvement consequent to practice.

### Procedure

In each experiment, we manipulated the type of practice, in two separate groups of randomly-assigned participants. In Experiment 1, one group received feedback while practicing *intensity* discrimination (supervised intensity practice), whereas a second group did not receive any feedback on performance (unsupervised intensity practice). In Experiment 2, one group received feedback while practicing *spatial* discrimination (supervised spatial practice), whereas a second group did not receive any feedback on performance (unsupervised spatial practice).

Each experiment was comprised of three sessions: one baseline session, one practice session (3 hours after baseline), and one testing session (3 hours after practice). In all groups, practice sessions lasted approximately 50 min. During supervised practice, participants performed either the intensity or the spatial discrimination task while receiving trial-by-trial feedback on performance (“correct”, “error”) by playing a pre-recoded robotic voice. Given that thresholds were estimated with the method of limits (see below), neither the number of trials within each practice block nor the number of practice blocks was fixed a priori.

A second experimenter blinded to the practice condition assessed the discrimination abilities of all participants at baseline and 3 hours after practice (+3 h test), each session lasting for approximately 1 hour. In each session, the blinded experimenter (K.D.) first tested detection thresholds for pinprick sensations, to ensure that thermal sensitivity was stable over the course of the day. Then, the blinded experimenter estimated two intensity discrimination thresholds, and two spatial discrimination thresholds, using the method of limits^10^,^48^. The order of presentation of intensity and spatial discrimination tasks was randomized across participants. During all sessions, subjects were blindfolded, and lay face down on a comfortable mattress.

To estimate the ***detection***thresholds for pinprick pain, we used the method of ascending staircases. In all experiments, we delivered slow-rising CO_2_ laser stimuli of increasing temperature (1 °C/sec). Participants were asked to press a button as soon as they felt a pinprick sensation, reflecting the activation of Aδ afferents^49^. The temperature at which the button was pressed defined the pinprick detection threshold. Two detection thresholds were measured in each subject, and were subsequently averaged.

To estimate the ***intensity discrimination*** thresholds, we delivered two consecutive stimuli with an inter-stimulus interval of 3 s. The two stimuli had different intensities but the same spatial location. The first stimulus was either more or less intense than the second stimulus, with equal probability of occurrence. The intensity of one of the two stimuli was fixed, and clearly detectable (47 °C; stimulus duration: 500 ms). To minimize effects of response bias, half of participants were asked to judge which stimulus in a pair was more intense, while the other half were asked to judge which stimulus was less intense. To avoid nociceptor fatigue, the laser beam was randomly shifted after each trial, within the defined 8 × 4 cm skin region in the low back. Thresholds were estimated using the method of limits. Blocks of ascending and descending staircases were alternated, and the first staircase type was balanced across participants. The initial intensity difference was 0.5 °C in ascending staircases, and 3 °C in descending staircases. The difference between the two stimuli was progressively adjusted, and the smallest stimulus difference was 0.5 °C. Intensity discrimination thresholds were defined as the minimal intensity difference that was correctly discriminated on three consecutive trials.

To estimate the ***spatial discrimination***thresholds, we delivered two consecutive stimuli with an inter-stimulus interval of 3 s. The two stimuli had identical intensities (47 °C), but different spatial locations. The first stimulus was either proximal or distal relative to the second stimulus, with equal probability of occurrence. To minimize effects of response bias, half of the participants of each group were asked to judge which stimulus in the pair was proximal, while the other half were asked to judge which stimulus was distal. To avoid nociceptor fatigue, the laser beam was randomly shifted after each trial, within the defined 8 × 4 cm skin region in the low back. Thresholds were estimated with the method of limits, following the same procedure described above and used in previous studies^10^. In ascending staircases, the initial distance between the two stimuli was 0.7 cm. In descending staircases, the initial distance was 5.6 cm. The distance between the two stimuli was progressively adjusted. The smallest distance between the two stimuli was 0.7 cm. Spatial discrimination thresholds were defined as the minimal distance at which the relative location of the two stimuli was correctly discriminated on three consecutive trials.

### Analyses

All t-tests were two-tailed, and *p*-values were estimated using a bootstrap procedure with 1,000 samples and 95% CI level. When the boostrapping procedure failed to estimate non significant p-values, this was indicated in the text as “bootstrap n/a”. We took into account the false discovery rate when interpreting p-values^50^.

## Additional Information

**How to cite this article**: Mancini, F. *et al*. Perceptual learning to discriminate the intensity and spatial location of nociceptive stimuli. *Sci. Rep.* **6**, 39104; doi: 10.1038/srep39104 (2016).

**Publisher's note:** Springer Nature remains neutral with regard to jurisdictional claims in published maps and institutional affiliations.
