# Corrigendum: Perceptual learning to discriminate the intensity and spatial location of nociceptive stimuli

**DOI:** 10.1038/srep42986

**Published:** 2017-03-07

**Authors:** Flavia Mancini, Karina Dolgilevica, James Steckelmacher, Patrick Haggard, Karl Friston, Giandomenico D. Iannetti

Scientific Reports 6: Article number: 39104; 10.1038/srep39104 published online: 12 20 2016; updated: 03 07 2017.

The original version of this Article contained an error in the spelling of the author Karina Dolgilevica, which was incorrectly given as Karina Dolgevica.

This error has now been corrected in the HTML and PDF versions of this Article.

